# Human footprint is associated with shifts in the assemblages of major vector-borne diseases

**DOI:** 10.1038/s41893-023-01080-1

**Published:** 2023-03-13

**Authors:** Eloise B. Skinner, Caroline K. Glidden, Andrew J. MacDonald, Erin A. Mordecai

**Affiliations:** 1 Department of Biology, Stanford University, Stanford, CA, USA; 2 Centre for Planetary Health and Food Security, Griffith University, Southport, Queensland, Australia; 3 Bren School of Environmental Science and Management, University of California, Santa Barbara, Santa Barbara, CA, USA; 4 Earth Research Institute, University of California, Santa Barbara, Santa Barbara, CA, USA

## Abstract

Predicting how increasing intensity of human–environment interactions affects pathogen transmission is essential to anticipate changing disease risks and identify appropriate mitigation strategies. Vector-borne diseases (VBDs) are highly responsive to environmental changes, but such responses are notoriously difficult to isolate because pathogen transmission depends on a suite of ecological and social responses in vectors and hosts that may differ across species. Here we use the emerging tools of cumulative pressure mapping and machine learning to better understand how the occurrence of six medically important VBDs, differing in ecology from sylvatic to urban, respond to multidimensional effects of human pressure. We find that not only is human footprint—an index of human pressure, incorporating built environments, energy and transportation infrastructure, agricultural lands and human population density—an important predictor of VBD occurrence, but there are clear thresholds governing the occurrence of different VBDs. Across a spectrum of human pressure, diseases associated with lower human pressure, including malaria, cutaneous leishmaniasis and visceral leishmaniasis, give way to diseases associated with high human pressure, such as dengue, chikungunya and Zika. These heterogeneous responses of VBDs to human pressure highlight thresholds of land-use transitions that may lead to abrupt shifts in infectious disease burdens and public health needs.

Humans have interacted with the environment and modified landscapes for millennia, but the rate of modification has accelerated in the past century^1^. Today, nearly 95% of the Earth’s terrestrial surface has been modified by humans, with almost 60% under intense or moderate pressure^1,2^. While anthropogenic environmental changes have cascading, and sometimes irreversible, impacts on natural and social systems^3^, we have only recently begun to quantify the extent and intensity of human pressure on a planetary scale^4,5^. Advances in satellite imagery, computational capacity and high-resolution data have led to the ability to map cumulative human pressure through time and space, opening the door to interdisciplinary applications for understanding the consequences of human pressures for human and planetary health.

When humans modify landscapes—either through large-scale conversion of natural habitat or in more localized and smaller-scale ways, such as hunting, selective logging and artisanal gold mining—they alter habitat structures and species interactions, each of which can shift the transmission of vector-borne diseases (VBDs)^6,7^. Recent decades have witnessed the emergence, re-emergence and geographic shifts of VBDs in many regions across the globe. These changes may have arisen from various human impacts on the environment and local ecology, including economic globalization, land use and climate change^8–13^. Arthropod disease vectors and the pathogens they transmit are highly sensitive to their environment, through a suite of traits that respond in complex, nonlinear and interactive ways^14,15^. Often, vectors and pathogens (that is, disease systems) occupy their own unique niche so that each transmission cycle responds distinctly to environmental change. With increasing anthropogenic pressure, one would expect transitions in the occurrence of different diseases; for instance, disease systems adapted to agricultural mosaics may be replaced by disease systems that thrive in urban sprawl. Yet, limited understanding of the relationships between different VBDs and human–environment interactions hinders projections of how disease assemblages collectively change across complex and changing landscapes^16^. The ability to anticipate these transitions would support a dynamic public healthcare infrastructure that can adapt to changes in disease occurrence through space and time.

One approach for investigating the effects of land-use change on VBD transmission uses broad classifications of land-use and land-cover classes, such as ‘urban area’ or ‘forest area’^17^. This is primarily because large-scale land conversions can be easily detected and monitored via space-borne satellites, and the detection of land-cover conversion is becoming ever more fine-scale. However, because pathogens respond to multidimensional features within a landscape (Supplementary Fig. 1), individual land-use classes are limited when predicting thresholds of change that may promote VBD occurrence. This is particularly challenging to quantify holistically across large land-use gradients and across different VBDs with unique ecologies. Moreover, assessments that use land-cover classes alone are inadequate to identify relationships between anthropogenic pressures on land and VBD transmission risks because they cannot always distinguish pressures that degrade, but do not outright convert, natural ecosystems^5,14^. This makes it difficult to separate the coupled natural and human processes that drive the association between specific land-use classes and disease.

Human footprint index (hereafter, human footprint) offers an ideal link between large-scale studies that use land-use classes and small-scale studies that have more detailed data because it is a single metric that captures the multidimensional influence of humans on land as it changes through space and time. Human footprint combines cumulative pressure mapping of eight indices at a fine spatial resolution (30 arcsec): (1) built environments, (2) population density, (3) electric infrastructure, (4) crop lands, (5) pasture lands, (6) roads, (7) railways and (8) navigable waterways^2,18,19^. Calculated as a continuous scale of increasing human pressure from 0 to 50, specific ranges of human footprint have been associated with variation in ecosystem function and integrity. Areas with human footprints of <4 are generally considered intact ecosystems that contain mostly natural habitat and maintain ecosystem integrity^2^. Studies on species extinctions have identified a human footprint threshold of ≥3 as a tipping point in which extinction events occur^20^. Areas with human footprints between 4 and 7 tend to be dominated by agricultural production, which exerts moderate to high human pressure on land^4^. Intense human pressure has been defined as areas with human footprints >12 (refs. ^4,20^). Since its early development in 2002, human footprint has been applied in a range of settings including biodiversity conservation^20,21^, climate change assessments^22,23^ and policy development^24^, but not to VBDs. Human footprint offers a unique opportunity to investigate thresholds of human pressure on VBDs across highly heterogenous environments that may promote or reduce disease risks at a broad geographic scale. In short, human footprint synthesizes pressures that might affect pathogen transmission and disease through multiple interrelated mechanisms, including changes in human mobility, vector (and reservoir host) habitat, contact between vectors and hosts, socioeconomic conditions and practices, and access to disease control measures and healthcare, among others.

We focus on Brazil as a case study of global patterns of human pressure and their relationships with VBDs because it is a large, ecologically and socio-economically diverse country that contains many biogeographic zones, intense and variable land-use pressures, a high incidence of multiple VBDs with contrasting ecologies and a long-standing nationwide disease surveillance system. Within Brazil we focus our analysis on the six most common VBDs of public health importance: dengue, chikungunya, malaria, Zika, cutaneous leishmaniasis and visceral leishmaniasis (Table 1). Aside from their public health importance, these diseases occur endemically within Brazil, are nationally notifiable and differ spatially and over time in patterns that probably reflect local socio-ecological conditions (Figs. 1 and 2). Our aim is to compare responses to human pressure among diseases with distinct ecologies, as a foundation for anticipating potential future disease responses to land-use change. Specifically, we test several key hypotheses: (1) land-use pressure and associated degradation, as captured by human footprint, is an important predictor of VBD occurrence (that is, whether or not at least one case is detected within a municipality in a given year); (2) the relationship with human footprint is nonlinear and differs predictably among VBDs on the basis of transmission ecology, with sylvatic and frontier diseases (malaria and cutaneous leishmaniasis) peaking at a lower human footprint than peri-urban and urban diseases (visceral leishmaniasis, dengue, chikungunya, Zika); (3) the predictive power of the human footprint on VBDs goes beyond that of total population size, capturing additional variation; (4) the suitability of other climatic factors such as temperature and rainfall promote VBD occurrence; and (5) in contrast to land-use classes alone, human footprint allows for easy-to-interpret comparisons of the response of multiple VBDs to human modified environments. Our goal is to understand the utility of human footprint as a predictor of VBDs with differing ecologies and to explore nonlinear relationships—in particular, threshold effects and transitions in disease assemblages across a gradient of landscapes—rather than to identify all possible predictors or to make causal inference, both of which are important future directions.

## Results and Discussion

Landscapes contain multiple VBDs, and while each system may respond to different individual features of a landscape, human footprint predicts disease occurrence across all six focal VBDs (mean importance = 10.46–44.91%, where the mean importance is the percent increase in standardized model error when the focal covariate is permuted). In the case of dengue and malaria, human footprint was greatly more important than any single land-use category (Fig. 3 and Supplementary Tables 1–7). As hypothesized, relationships between human footprint and local pathogen transmission were nonlinear and varied in direction among pathogens (Fig. 4a and Supplementary Table 8). Specifically, we found that human footprint had an increasing relationship with disease occurrence for the urban diseases (dengue, chikungunya and Zika) and a decreasing relationship for the sylvatic or frontier diseases (malaria and cutaneous leishmaniasis). The relationship of visceral leishmaniasis, a formerly rural disease now expanding into peri-urban areas^25^, with human footprint was a hybrid of the responses of urban and sylvatic diseases, steeply declining with a human footprint between 8 and 17 then increasing with a human footprint above 17. Our results provide a basis for comparing shifts in VBD assemblages across landscapes, rather than focusing on single pathogens.

The distinct threshold responses across VBDs highlight the need for policies that account for the potentially varied impacts of human pressure on pathogen transmission. Specifically, while this analysis is descriptive and does not explicitly capture changes over time, we found that gradients in human footprint correspond to gradients in the occurrence of different diseases, which require distinct control strategies. Supporting this idea, all of the focal diseases that have been present in Brazil since the early twenty-first century have shifted in distribution and incidence in the past decade (Supplementary Fig. 2); however, comparable human footprint data with which to directly test this prediction are not available for the historical period.

Pathogen transmission is a complex process that responds to multiple aspects of environmental change^14^. For example, climate, land use, ecosystem transitions and mobility affect the probability of humans encountering infectious vectors, whose presence in turn depends on environmentally sensitive factors such as vector abundance, contact with infectious reservoirs and vector competence. Identifying human footprint thresholds can help predict tipping points at which human activities might lead to qualitative changes in disease risk. Specifically, our results show that areas that have undergone high to intense human pressure (a human footprint between 6 and 14) are more likely to harbour diseases transmitted by the urban mosquito *Aedes aegypti* (dengue, chikungunya and Zika) and less likely to harbour the more sylvatic and rural diseases (malaria, cutaneous leishmaniasis and visceral leishmaniasis) (Fig. 4a and Supplementary Table 8). The relative importance of human footprint and the estimated human footprint transition threshold differs among pathogens, probably reflecting their unique disease ecology (Figs. 3 and 4). For example, the probability of malaria occurring in Brazil steeply declines at a human footprint above 5, which is associated with transitions between intact ecosystems and intensive agricultural practices, supporting previous findings that malaria transmission increases with frontier forest clearing^26^. By contrast, dengue, chikungunya and Zika steadily increase in occurrence with human footprint, and their probability of occurrence is maximized at a human footprint above 8–12. These values correspond to intense human pressure, including built environments, high population density and extensive transportation networks (such as roads, railways or navigable waterways). These urban environments are established habitats for populations of *Ae. aegypti*, the primary vector of dengue, chikungunya and Zika in Brazil^27,28^.

Total population was the most important predictor of occurrence for all VBDs except dengue, for which human footprint and temperature were equally important (Fig. 3). Using both human footprint and total population, which were not strongly correlated (correlation coefficient *r* = 0.30) (Supplementary Fig. 3), in a single model allowed us to distinguish the impact of humans on the land from the impact of the total population. Urbanization was not included in the model as it is the most highly weighted contributor to calculations of human footprint and thus was strongly correlated with human footprint^2^. Unlike human footprint, which showed nuanced and nonlinear impacts that qualitatively differed by VBD ecology, population size had a monotonically increasing relationship with probability of disease occurrence (Supplementary Fig. 4). This positive relationship was expected because larger populations have a greater number of susceptible hosts, a higher probability of introduction and an increased capacity for disease detection and reporting.

**Fig. 1 |:**
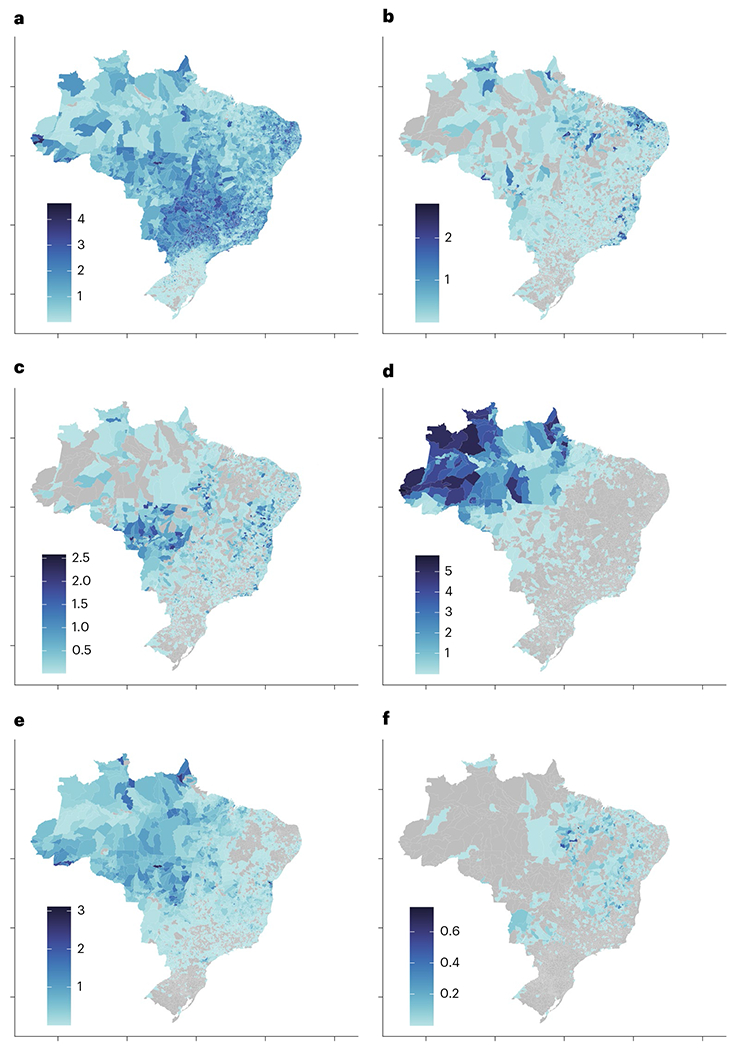
Disease incidence for the six VBDs. **a**–**f**, Log-transformed average annual cases per 1,000 per municipality (colour scale) for dengue (**a**), chikungunya (**b**), Zika (**c**), malaria (**d**), cutaneous leishmaniasis (**e**) and visceral leishmaniasis (**f**). The average incidence is shown here for illustration, but models are based on the binary occurrence of each disease within each municipality per year (municipality–year level response). The averages are calculated from 2013 to 2019 in **a,d**–**f**, from 2017 to 2019 in **b** and from 2016 to 2019 in **c**. Grey municipalities did not report any cases of the disease during the study period.

The relationships of Zika and visceral leishmaniasis to human footprint merit further discussion. Although human footprint greatly improved model performance, for these two pathogens, human footprint was equal to but not better than land-class categories in importance for predicting occurrence (Fig. 3). Furthermore, in other land-class studies, Zika transmission has been associated with cropland and grassland areas^29^, but here we found the measure of footprint threshold (the value at which Zika reaches 50% of its highest probability of occurrence) to be the highest (human footprint = 12.89 with 95% CI of 12.82 to 12.96) of any selected VBD. The recent introduction of Zika into Brazil in 2015 may play a role in this discrepancy because the explosive, country-wide epidemic was probably driven by high host susceptibility and stochastic effects of early introductions^30^, and may not fully represent the equilibrium socio-environmental conditions associated with endemic transmission. A visual comparison with spatial patterns of chikungunya incidence (Fig. 1) supports this point, as the pathogens share a vector and socio-ecological conditions underlying transmission, yet had distinct incidence patterns that may reflect the impact of stochasticity during emerging epidemics. It is therefore likely that, while Zika occurrence increases with human footprint, epidemic case data from Brazil exaggerate this relationship due to the high susceptibility of human population and the explosive spread of emerging pathogens in urban areas^31^.

**Fig. 2 |:**
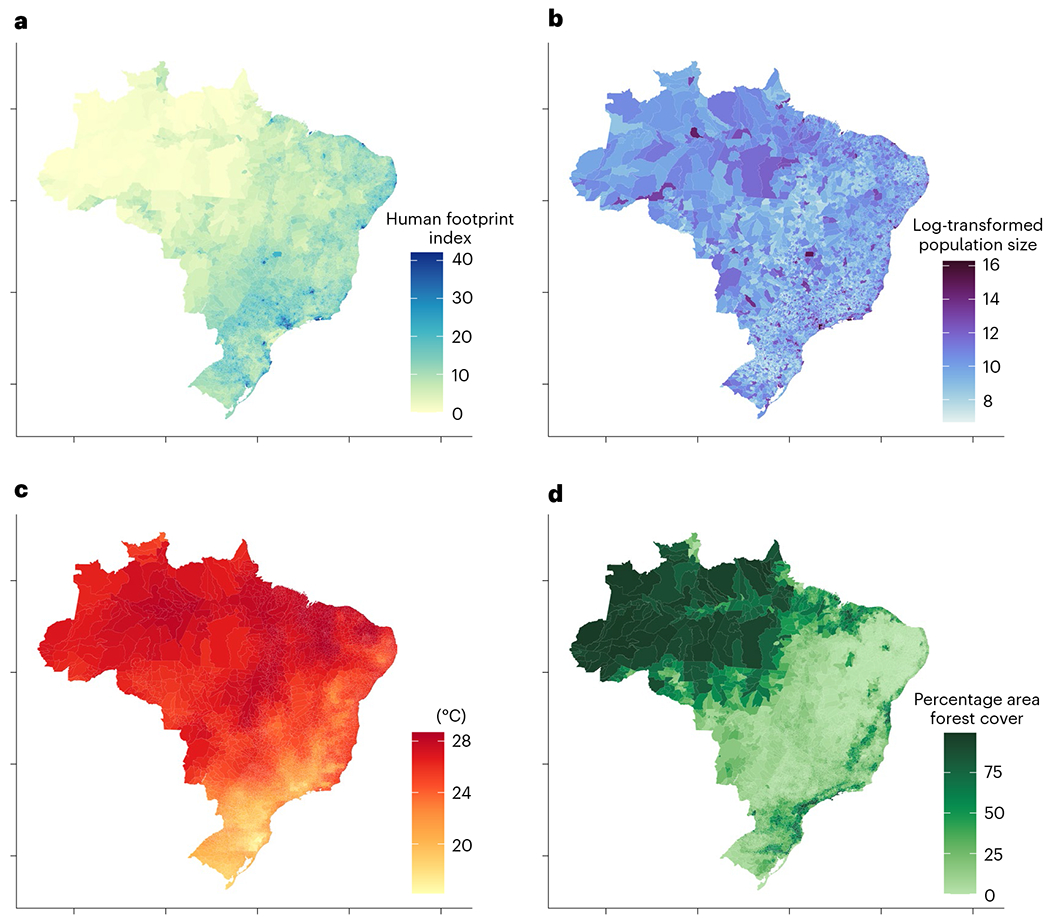
Environmental covariates that predict VBD occurrence in Brazil averaged from 2013 to 2019. **a**, Human footprint, ranging from 0 to 50, for which values from 4 to 7 indicate moderate pressure (typical for agricultural landscapes) and values greater than 12 indicate intense pressure. **b**, Log-transformed human population size. **c**, Average annual temperature. **d**, Percentage area forest cover.

**Fig. 3 |:**
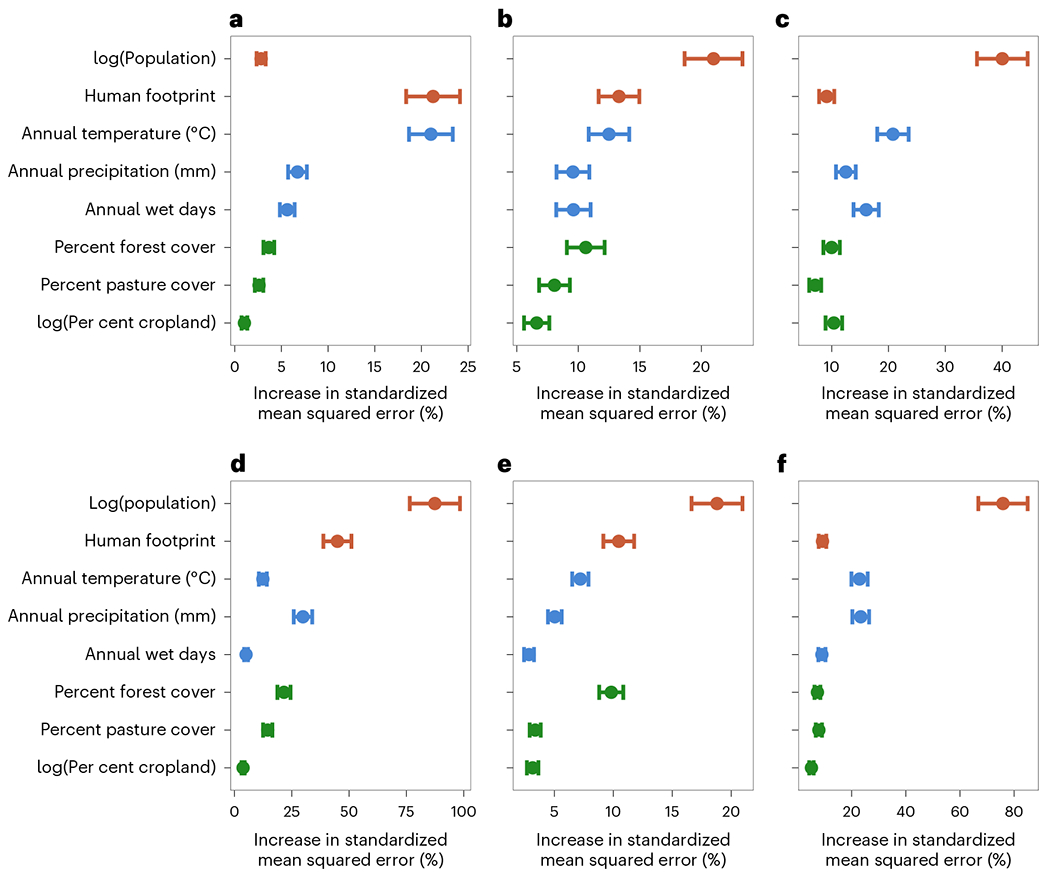
The importance of environmental predictors of the occurrence of the six VBDs. **a**–**f**, The contribution of variables to the prediction accuracy of the overall model (multiplied by 100 for visualization) for dengue (**a**), chikungunya (**b**), Zika (**c**), malaria (**d**), cutaneous leishmaniasis (**e**) and visceral leishmaniasis (**f**). The point represents the mean % change in model standard error when the selected variable is permuted, the error bars represent the 95% confience interval across subsampling permutation iterations. The variables are colour coded according to their categories (anthropogenic, climatic and land-class variables are shown in red, blue and green, respectively).

**Fig. 4 |:**
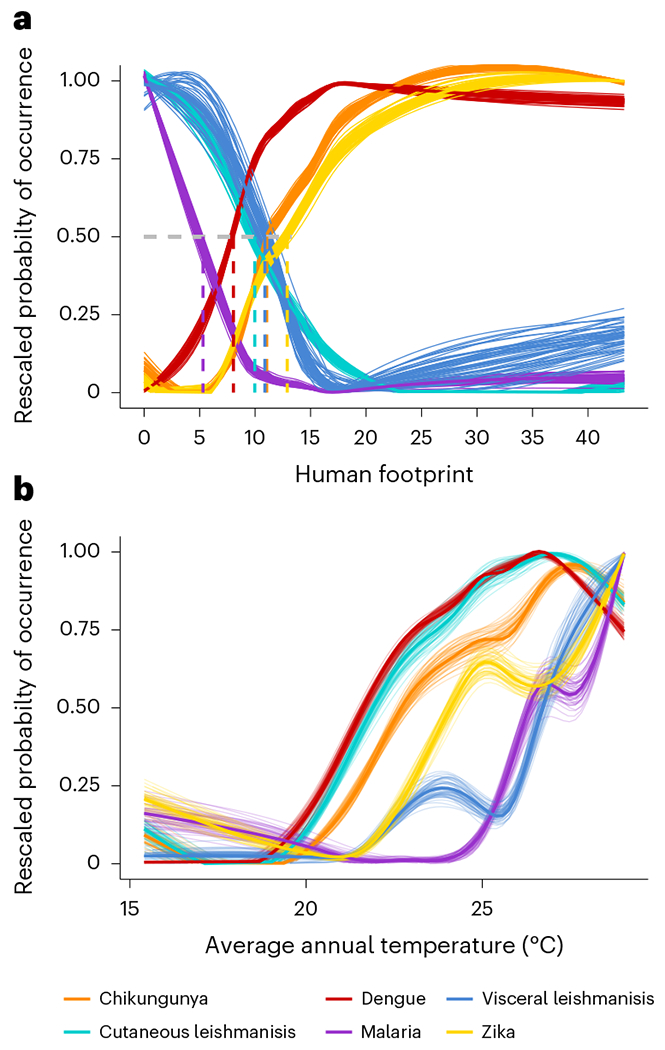
Probability of disease occurrence across human footprint and temperature. **a**,**b**, VBDs respond distinctly and nonlinearly to human footprint (**a**) and average annual temperature (**b**). Thin lines represent model output from each bootstrapped iteration, and thicker lines represent the mean value across the iterations. The dashed lines in **a** represent the threshold value at which a pathogen reaches 50% of its maximum occurrence probability.

Visceral leishmaniasis had a non-monotonic relationship with human footprint, such that the probability of visceral leishmaniasis occurrence initially decreased and then increased with higher human footprint values, which was overall relatively less important than for the other focal VBDs (Figs. 3 and 4). By contrast, we had hypothesized that, as a peri-urban disease that cycles between sandflies, domestic dogs and humans, visceral leishmaniasis would monotonically increase with human footprint. It is possible that the observed relationship is a result of the low overall incidence and geographic range of visceral leishmaniasis, which may be confounded with spatial patterns of human footprint (Figs. 1 and 2). Alternatively, it is possible that human footprint does not capture the multiple socio-environmental conditions involved in visceral leishmaniasis transmission ecology as well as it does for other VBDs. Recent work in the Brazilian state of São Paolo has found a link between deforestation and visceral leishmaniasis as the disease has spread from formerly endemic rural areas into urban areas in conjunction with the development of an oil pipeline^25^. As for most of the other focal VBDs, human population was very important for predicting visceral leishmaniasis occurrence, and in this case may represent the joint effect of human population on the availability of susceptible humans and dogs. The Ministry of Health estimates that there is one dog for every four people in Brazil, suggesting a strong linear relationship between human population size and the number of reservoir hosts^32^.

In addition to the impacts of human footprint and population size, we expected climate to play an important role in determining VBD transmission because temperature, precipitation and humidity are known to constrain vector distributions and biology^33,34^. We hypothesized that temperature would have a nonlinear, increasing relationship with the transmission of all six VBDs. All focal pathogens showed a positive relationship with average annual temperature that increased steeply between 20 °C and 27 °C, indicating an important temperature threshold for disease occurrence. Dengue, cutaneous leishmaniasis and chikungunya had relatively lower threshold temperatures, reaching 50% of their maximum occurrence probabilities at 22–23 °C, while Zika, malaria and visceral leishmaniasis reached 50% of their maximum occurrence probability at higher temperatures of 25–27 °C (Fig. 4b). Temperature directly affects vector ecology, competence and parasite infectivity, and our results support its importance for all six focal VBDs (Fig. 3). Identifying more precise differences in thermal responses of diseases (for example, ref. ^33^) from this kind of observational study is challenging because climate is confounded by the geographic associations of diseases with land use and other factors. For example, malaria in this region is largely restricted to the Amazon rainforest, which is closer to the Equator and therefore warmer than other parts of Brazil. Rainfall was also an important predictor for all six VBDs (Fig. 3), as expected from the reliance of vectors on standing water and/or humid habitats for breeding.

Accelerating rates of land conversion, human population growth, demand for resources and climate change make it essential to identify thresholds of human pressures that correspond to increased or decreased risk of VBD transmission. Here we compared the multidimensional relationship between human activities, measured as human footprint, with the transmission of multiple VBDs at a broad geographic scale using a machine learning approach (Methods and Supplementary Fig. 5). This study is a first large-scale test of the hypothesis that human footprint has multidimensional, distinct and nonlinear effects on VBDs that mirror their transmission ecology, and our findings support this hypothesis.

There are several important limitations to this approach that future work should address. First, this study is observational and does not necessarily capture underlying causal mechanisms. It is subject to under-reporting of disease cases (particularly for pathogens that frequently cause asymptomatic infection) and measurement error and autocorrelation among environmental covariates. Second, while human footprint represents an important advance in globally accessible, high-resolution mapping of multidimensional impacts of humans on landscapes, it is currently only available for recent time periods and is updated infrequently. As a result, we had to interpolate between 2013 and 2019 and across two methods (the original computation and a validated machine learning method^2,18^) to calculate human footprint for each study year. This is limiting because the assumption of a constant, linear change in human footprint from year to year probably biases our results to be conservative because we are not able to catch year-specific shocks in human pressure that could result in rapid changes in VBD occurrence. We also hypothesize that the rate at which a municipality changes in human footprint from one year to the next is important for disease occurrence and, if available, would better define the tipping points in our partial dependence plots (PDPs) and reduce the uncertainty in our variable importance measures. Third, the socio-ecological predictors of disease occurrence may not be the same as the drivers of outbreak size, so the areas that have a high probability of disease occurrence are not necessarily those with the highest disease risk or burden. In particular, disease incidence can vary substantially due to variation in susceptible host population size, vector control measures and access to healthcare and other services. Our primary goal was to capture the land-use niches of multiple VBDs in a comparative approach and to identify critical thresholds across which a more intense human footprint could lead to shifts in disease occurrence. Important directions for future work include conducting causal analyses to understand whether shifts across human footprint thresholds lead to shifts in VBD occurrence (and at what timescales) and investigating how human pressure interacts with socioeconomic variables as drivers of VBD incidence.

Comparing six important VBDs in Brazil, we found that human footprint is an important predictor of local occurrence and that its nonlinear effects vary predictably with the transmission ecology of each VBD. In a critical window in which human footprint changes from moderate (4–7) to high (7–12) to intense (>12), disease occurrence abruptly shifts from malaria, cutaneous leishmaniasis and visceral leishmaniasis to dengue, chikungunya and Zika (arboviruses transmitted by the urban mosquito *Ae. aegypti* and diseases that require distinct responses in vector control, diagnostics and environmental management). Because biomedical and chemical approaches alone have failed to sustainably eliminate these VBDs, managing the socio-ecological settings that promote pathogen transmission is a critical frontier for planetary health. In conjunction with climatic pressures, human pressure presents a major risk for disease emergence and transmission, threatening the well-being of humans and the environment.

## Methods

### Study location and VBDs

We focused our analysis on six endemic VBDs in Brazil. The diseases are associated with a significant global health burden (collectively estimated to cause more than 620 million cases worldwide annually) but vary in their vector and reservoir host ranges and their hypothesized ecological and climatic niches. Moreover, the six VBDs encompass a range of diseases classically considered to be frontier, rural or urban. As a result, we expected variations in their responses to Brazil’s large range of human footprint. We initially included yellow fever virus as a seventh disease, but extreme data sparsity (only 64 cases reported throughout the study period) restricted analysis of this pathogen. Brazil is an ideal country in which to test environmental predictors of VBDs because (1) data on infections are publicly available for more than 5,500 municipalities, (2) Brazil has diverse and concentrated land-use types and anthropogenic pressures (for example, ranging from pristine forests to intensive agricultural production and high-density cities), (3) Brazil spans a range of climatic conditions, from equatorial tropical conditions to more temperate conditions in the south of the country.

### Data collection and preparation

For all diseases except malaria, annual case data were collected from the Brazilian national disease surveillance system (SINAN) for each municipality from 2013 to 2019^35^. For malaria, disease notification data were collected from the Brazil Epidemiological Surveillance Information System for Malaria (SIVEP–MALARIA) for two parasites (*Plasmodium vivax* and *Plasmodium falciparum*) for each municipality and year^36^. While we collected and mapped data on case incidence (Fig. 1), we modelled disease occurrence (binary: whether or not a disease occurs in a municipality in a given year) here to capture the ecological niche for each disease with respect to land use and other environmental variables. The dengue, malaria, visceral leishmaniasis and cutaneous leishmaniasis analyses included 38,893 municipality–year observations; Zika included 22,276 municipality–year observations (reported in 2016–2019, inclusive); and chikungunya included 16,707 municipality–year observations (reported in 2017–2019, inclusive).

Human footprint was included as the primary measure of anthropogenic pressure. We assumed that the probability of pathogen occurrence directly increases with population size and expected socioeconomic factors to influence rates of transmission through multiple pathways (for example, housing quality, investment in control measures, education and awareness of disease risk factors). However, we were primarily interested in landscape changes and environmental degradation, as these factors were expected to strongly influence the ecology of the focal vector species and the pathogens they transmit and how human populations interact with and use land; they may also be broadly predictive of disease occurrence across both ecological and socioeconomic contexts. Therefore, to isolate landscape level changes of human footprint, we included human population as a covariate in our models. Human footprint is a global, dimensionless index of human pressure on the land surface and is calculated from eight different human pressures: (1) built environments, (2) population density, (3) electric infrastructure, (4) crop lands, (5) pasture lands, (6) roads, (7) railways and (8) navigable waterways. The average annual human footprint for each municipality was calculated from two datasets: the estimate for 2013^2^ and an updated machine learning estimate for 2019^18^, both of which calculated human footprint at 1 km spatial resolution. The average human footprint for each municipality was extracted for 2013 and 2019 using a shapefile with municipality boundaries in R (version 4.0.2) (ref. ^37^). The extracted values were then interpolated for the years 2014–2018, assuming a constant rate of change. Population data per municipality per year from 2013 to 2019 were extracted from the WorldPop database^38^.

Temperature, precipitation and humidity are known to mediate the transmission of VBDs through several mechanisms including reproduction, development, behaviour and population dynamics^33,34^. For our analysis, we were interested in climate variables that constrain vector biology but also capture variation between municipalities on an annual scale. We originally considered annual mean temperature, cumulative precipitation, the total number of wet days climate extremes (minimum temperature, maximum temperature, minimum precipitation, maximum precipitation) and climate variance (temperature seasonality). There was high correlation, however, between many of these variables, and only the annual mean temperature, cumulative precipitation and the total number of wet days were used in the final analysis. Climate data were extracted from the Climate Research Unit, a global gridded satellite dataset with a resolution of 0.5° × 0.5° (ref. ^39^) (Fig. 2c and Supplementary Fig. 6a,b). Average climate data were extracted using a shapefile of municipality boundaries in R, for each municipality and year from 2013 to 2019.

Three land-class categories were included in the analysis: pasture, cropland and forest area (Fig. 2d and Supplementary Fig. 6c,d). For each municipality, the total area of each land-class category from 2013 to 2019 was collected from MapBiomas^40^, a network that produces annual land-use and land-class maps for Brazil using Google Earth Engine cloud computing technology to process Landsat data. The percentage cover for each of the three categories was then calculated from the total area of the municipality for each year. Percentage cropland areas were log-transformed for normalization. While we included per cent cover of major land-use and land-cover categories (forest, cropland, pasture), we excluded urban cover. Urbanization is a highly weighted factor in human footprint, which includes built environment, night-time lights and the density of roads. However, because human footprint also includes information on population density, crop and pasture cover, railways and navigable rivers, our central argument here is that it more holistically captures the multidimensional effects of human pressure that may be related to VBD transmission, and therefore is a better predictor than urban cover alone.

### Statistical analysis

The temporal resolution of human footprint and land-cover data limited our analysis to an annual scale. The analysis methods are summarized in Supplementary Fig. 2. Briefly, we used a machine learning approach to assess the relationships between human footprint, climate, land-class categories and vector presence and the occurrence of six VBDs in Brazil. Machine learning approaches are increasingly being applied in disease ecology because they can accommodate complexity and nonlinearity to identify linkages among environmental factors and disease^41^. To understand the predictors of occurrence for each VBD, we used a random forest model, a versatile machine learning technique that uses randomized recursive partitioning to solve complex prediction, regression and classification problems. These models work by repeatedly drawing bootstrap samples from the original sample and a random selection of predictors to grow a predetermined number of decision trees across which results are pooled^42^.

Although collinearity does not influence random forest model performance, it can distort the magnitude of the variable importance and complicate the interpretation of variable response curves^43^. The direction and strength of relationships between explanatory variables was assessed using Pearson’s correlation and Spearman tests and calculated using the package corrplot in R^44^. We considered a correlation coefficient of >0.7 to indicate high correlation between variables^45^ and removed one of the two correlated variables based on a priori empirical knowledge. Ultimately, we excluded climate extremes (minimum temperature, maximum temperature, minimum precipitation, maximum precipitation), climate variance (temperature seasonality) and percentage urban cover.

Once the full dataset had been cleaned and compiled, the modelling process involved the following steps:

- Explanatory variables: all environmental predictors were included in each model, except in the supplementary model that includes vector occurrence (Supplementary Fig. 7), in which only the presence of the most relevant vector was used for each disease (for example, the presence of *Anopheles* was only used in the malaria models) (Supplementary Table 9).
- Model training, tuning, selection: for each model iteration, the optimal number of variables randomly sampled as candidates for each split (mtry) and node size was calculated using the tune function in the randomforestSRC package in R^46^. The tune function estimated model performance (out-of-bag error) for a range of combinations of mtry and node size values and selected the optimal values as the combination that yielded the lowest model error. All models were grown to 500 trees to maximize model performance and minimize computational costs.
- Model analysis: classification analysis was performed with the imbalance function in the randomforestSRC package, using the random forest quantile-classifier method^47^.
- Model validation: spatiotemporal cross-validation was used to evaluate model performance to better understand the predictive power of the model in geographic regions and years not used for training. Municipalities in Brazil were split geographically into 15 folds using the R package spatialsample^48^. The folds were assigned using *k*-means clustering, in which each municipality belongs to a cluster with the nearest mean centroid. The data were then split into three expanding windows: (1) data from 2013 to 2016 were used in the training set, and data from 2017 were used as the test set, (2) data from 2013 to 2017 were used for training and data from 2018 were used for testing, (3) data from 2013–2018 were used for training and data from 2019 were used for testing. Within each window, the model was then tuned and trained 15 times, where, for each iteration, 14 folds were used to train the model, and the hold-out fold was used to evaluate model performance (see below). In summary, the test set only contained municipalities that were unique in both space and time in comparison to the training set. In total, for all pathogens except chikungunya, the model was trained and tested 45 times. For chikungunya, which was detected in Brazil starting in 2017, the model was trained and tested 30 times.

We calculated three groups of metrics from the random forest models for each pathogen. First, we assessed overall model performance by calculating model sensitivity, specificity and the area under the receiver operating characteristic curve based on the spatiotemporal cross-validation. For the out-of-sample model performance metric, we calculated the mean and 95% confidence interval around each of these values.

Second, we calculated variable importance, which can be interpreted as the contribution of each explanatory variable to the prediction accuracy of the model using the computationally optimized ‘random’ algorithm in the randomforestSRC package. The contributions to the prediction accuracy were estimated by calculating the per cent increase in the standardized mean squared error when the focal variable was permuted. To test whether each variable significantly contributed to model accuracy, we conducted a permutation-based significance test using a subsampling method. This estimated the probability (permutation-based *P* value) that the change in model accuracy when the covariate is permuted versus not permuted was greater than or equal to 0 (that is, that the null hypothesis is supported).

Finally, we assessed the overall form and direction in which each explanatory variable relates to disease occurrence using PDPs. PDPs depict the relationship between the probability of disease occurrence and the variable of interest across a range of values for that feature. At each value of the feature, the model was evaluated for all values of the other covariates; the final model output was the average predicted probability across all model inputs. The data frame underpinning the PDPs was constructed using the plot.variable function in the randomforestSRC package in R, and the relationships between each feature and the predicted probabilities were plotted with ggplot2, using a loess transformation (that is, locally weighted smoothing). To compare the qualitative shape of PDPs across pathogens, the PDPs in the main text were scaled by their minimum and maximum and plotted on a single plot. We defined a threshold as the value at which a pathogen reaches 50% of its maximum occurrence probability. As a result, these scaled PDPs should be interpreted as comparing the direction and nonlinearity of responses of different diseases to environmental predictors, but not their absolute magnitude or relative importance (which is conveyed in variable importance plots). Unscaled PDPs are presented in Supplementary Figs. 8–11. To generate measures of uncertainty around our estimates of the relationship between each covariate and pathogen occurrence, we used a bootstrapping approach where the model was iterated 50 times using different subsets of 80% of the full dataset. Our results displayed the average relationship across model iterations as well as a PDP per iteration.

### Reporting summary

Further information on research design is available in the Nature Portfolio Reporting Summary linked to this article.

## Supplementary Material

supplement

## Figures and Tables

**Table 1 |:** Selected VBDs and their associated land-use categories, vectors, vertebrate hosts and global burden

VBD	Associated land-use types	Disease vector(s)	Vertebrate hosts	Global burden
Dengue	Urban^28^	*Aedes aegypti, Aedes albopictus*	Humans	390 million cases yr^−1^ (ref. ^49^)
Chikungunya	Urban, peri-urban^50^	*Ae. aegypti, Ae. albopictus*	Humans	330,000 cases yr^−1^ (ref. ^51^)
Malaria	Forest edge, agriculture^52^	*Anopheles spp.*	Humans	229 million cases yr^−1^ (ref. ^13^)
Zika	Urban, rural, peri-urban^29^	*Ae. aegypti, Ae. albopictus*	Humans	100,000 cases yr^−1^ (ref. ^11^)
Cutaneous leishmaniasis	Forest, rural^53,54^	>60 sandfly species	Humans, rodents, xanthra, marsupials, primates	600,000–1,000,000 cases yr^−1^ (ref. ^55^)
Visceral leishmaniasis	Rural, peri-urban, urban^56^	*Lutzomyia longipalpis*	Humans, domestic dogs	50,000–90,000 cases yr^−1^ (ref. ^55^)

## Data Availability

All datasets that have been used for this study are publicly available and links have been provided for each within the Methods and in the GitHub repository at https://github.com/ckglidden/human-footprint-index-VBD. Source data are provided with this paper.
