# Supplementary material for: Human footprint is associated with shifts in the assemblages of major vector-borne diseases
Source: Nat Sustain. Author manuscript; Available in PMC 2023 Aug 3. (PMC10399301; doi:10.1038/s41893-023-01080-1)
Supplement: supplement [file NIHMS1914402-supplement-supplement.pdf]

nature sustainability

Article https://doi.org/10.1038/s41893-023-01080-1

Human footprint is associated with shifts in the assemblages of major vector-borne diseases

In the format provided by the authors and unedited

Supplementary Tables and Captions

Supp. Table 1: Model performance metrics for each focal disease; sensitivity, specificity, and AUC for out-of-sample model performance (spatiotemporal cross-validation). Out-of-sample metrics include the mean and 95% confidence intervals across 30 folds for chikungunya and 45 folds for all other pathogens (divided using an expanding time window and k-folds clustering).

**Pathogen**	**Sensitivity**	**Specificity**	**AUC**
Dengue	0.61 (0.56-0.65)	0.67 (0.62-0.72)	0.72 (0.70-0.76)
Chikungunya	0.63 (0.58-0.68)	0.78 (0.74-0.82)	0.74 (0.72-0.77)
Zika	0.64 (0.59-0.69)	0.76 (0.73-0.80)	0.73 (0.70-0.76)
Malaria	0.82 (0.78-0.86)	0.82 (0.79-0.85)	0.85 (0.83-0.88)
Visceral leishmaniasis	0.83 (0.79-0.88)	0.81 (0.77-0.84)	0.85 (0.82-0.88)
Cutaneous leishmaniasis	0.65 (0.61-0.70)	0.76 (0.72-0.79)	0.73 (0.70-0.76)

Supp. Table 2. Mean and 95% CI for variable importance, on a scale of 0-100, for the dengue model using the subsampling procedure and delete-d jackknife estimators of the variance implemented by the R package *randomForestSRC*. P-value is based on a permutation test.

	lower	mean	upper	p-value
human footprint index	18.46	21.25	24.03	<0.001
log(% cropland)	0.71	1.04	1.37	<0.001
% forest	3.06	3.65	4.25	<0.001
% pasture	2.13	2.61	3.08	<0.001
log(population size)	2.39	2.83	3.26	<0.001
annual temperature	18.55	21.01	23.47	<0.001
annual precipitation	5.76	6.72	7.68	<0.001
annual no. wet days	4.83	5.63	6.42	<0.001

Supp. Table 3. Mean and 95% CI for variable importance, on a scale of 0-100, for the chikungunya model using the subsampling procedure and delete-d jackknife estimators of the variance implemented by the R package *randomForestSRC*. P-value is based on a permutation test.

	lower	mean	upper	p-value
human footprint index	11.73	13.30	14.87	<0.001
log(% cropland)	5.64	6.62	7.60	<0.001
% forest	9.07	10.60	12.14	<0.001
% pasture	6.86	8.07	9.27	<0.001
log(population size)	18.70	20.98	23.26	<0.001
annual temperature	10.80	12.49	14.18	<0.001
annual precipitation	8.26	9.56	10.86	<0.001
annual no. wet days	8.23	9.60	10.98	<0.001

Supp Table 4. Mean and 95% CI for variable importance, on a scale of 0-100, for the Zika model using the subsampling procedure and delete-d jackknife estimators of the variance implemented by the R package *randomForestSRC*. P-value is based on a permutation test.

	lower	mean	upper	p-value
human footprint index	7.82	9.12	10.42	<0.001
log(% cropland)	8.89	10.38	11.87	<0.001
% forest	8.50	9.99	11.47	<0.001
% pasture	5.96	7.10	8.24	<0.001
log(population size)	35.46	40.03	44.59	<0.001
annual temperature	18.01	20.78	23.54	<0.001
annual precipitation	10.74	12.50	14.25	<0.001
annual no. wet days	13.81	16.07	18.33	<0.001

Supp Table 5. Mean and 95% CI for variable importance, on a scale of 0-100, for the malaria model using the subsampling procedure and delete-d jackknife estimators of the variance implemented by the R package *randomForestSRC*. P-value is based on a permutation test.

	lower	mean	upper	p-value
human footprint index	38.89	44.91	50.93	<0.001
log(% cropland)	3.16	3.75	4.35	<0.001
% forest	18.71	21.59	24.48	<0.001
% pasture	12.55	14.51	16.47	<0.001
log(population size)	76.27	87.41	98.55	<0.001
annual temperature	10.63	12.40	14.15	<0.001
annual precipitation	25.82	29.87	33.93	<0.001
annual no. wet days	4.23	5.06	5.89	<0.001

Supp Table 6. Mean and 95% CI for variable importance, on a scale of 0-100, for the cutaneous leishmaniasis model using the subsampling procedure and delete-d jackknife estimators of the variance implemented by the R package *randomForestSRC*. P-value is based on a permutation test.

	lower	mean	upper	p-value
human footprint index	9.07	10.46	11.84	<0.001
log(% cropland)	2.61	3.16	3.70	<0.001
% forest	8.80	9.82	10.85	<0.001
% pasture	2.87	3.37	3.87	<0.001
log(population size)	16.64	18.79	20.95	<0.001
annual temperature	6.46	7.20	7.94	<0.001
annual precipitation	4.41	5.04	5.66	<0.001
annual no. wet days	2.40	2.85	3.30	<0.001

Supp Table 7. Mean and 95% CI for variable importance, on a scale of 0-100, for the visceral leishmaniasis model using the subsampling procedure and delete-d jackknife estimators of the variance implemented by the R package *randomForestSRC*. P-value is based on a permutation test.

	lower	mean	upper	p-value
human footprint index	7.77	9.24	10.72	<0.001
log(% cropland)	4.24	5.16	6.09	<0.001
% forest	6.24	7.37	8.51	<0.001
% pasture	6.70	7.90	9.11	<0.001
log(population size)	66.77	75.88	85.00	<0.001
annual temperature	19.97	22.93	25.89	<0.001
annual precipitation	20.24	23.37	26.49	<0.001
annual no. wet days	7.68	9.02	10.36	<0.001

Supp. Table 8. Mean inflection points (human footprint index when scaled probability is 50%) and 95% confidence intervals across 50 model iterations, with each iteration fit to a unique subset of 80% of the data.

**Pathogen**	**Value at 50% scaled probability**	**Increasing at inflection point?**
Dengue	8.05 (8.02 – 8.07)	increasing
Chikungunya	11.04 (11.00 – 11.09)	increasing
Zika	12.89 (12.82 – 12.96)	increasing
Malaria	5.30 (5.25 – 5.36)	decreasing
Visceral leishmaniasis	10.88 (10.76 – 10.98)	decreasing
Cutaneous leishmaniasis	9.97 (9.90 – 10.04)	decreasing

Supp. Table 9. Proven vectors of cutaneous leishmaniasis, with occurrence points on GBIF that were used in the cutaneous leishmaniasis model.

**Sandfly species**
Bichromomyia flaviscutellata
Lutzomyia (Helcocyrtomyia) hartmanni
Lutzomyia (Tricholateralis) gomezi
Migonemyia (Migonemyia) migonei
Nyssomyia anduzei
Nyssomyia intermedia
Nyssomyia intermedia
Nyssomyia neivai
Nyssomyia umbratilis
Nyssomyia whitmani
Nyssomyia yuilli
Pintomyia (Pifanomyia) spinicrassa
Pintomyia (Pintomyia) fischeri
Pintomyia nuneztovari
Psathyromyia (Psathyromyia) shannoni
Psychodopygus ayrozai
Psychodopygus carrerai
Psychodopygus complexus
Psychodopygus llanosmartinsi
Psychodopygus panamensis
Psychodopygus squamiventris maripaensis
Psychodopygus thula
Psychodopygus wellcomei
Trichophoromyia ubiquitalis
Lutzomyia longipalpis

Supplementary Figures and Captions

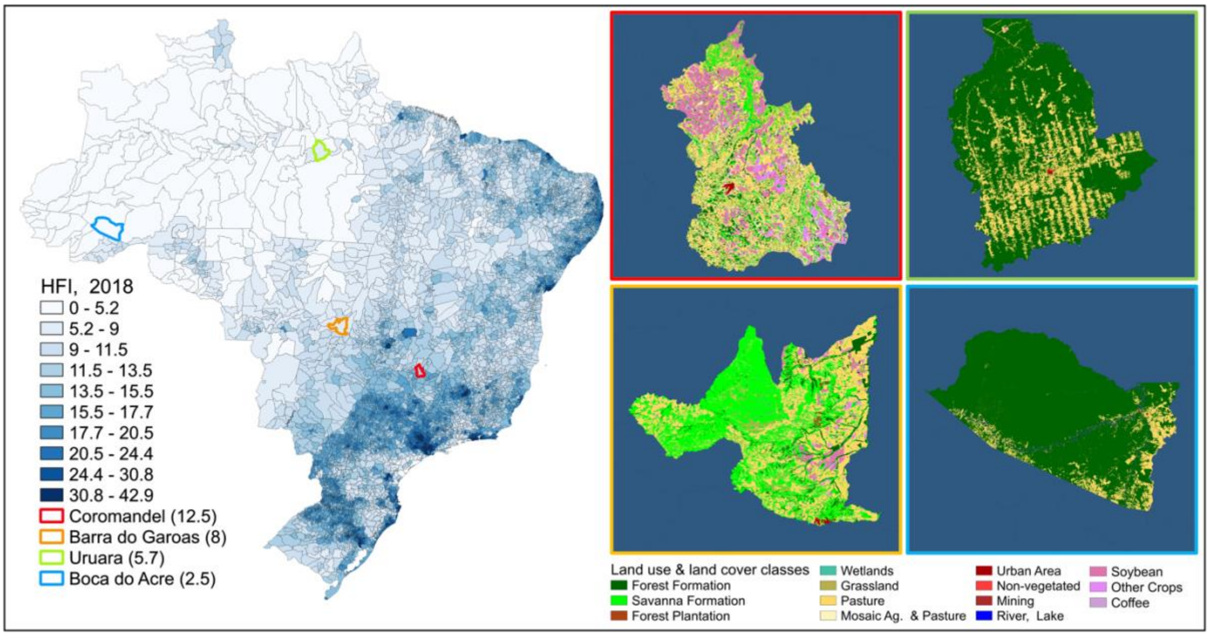

Supp. Fig. 1. Land use and land cover classifications (from MapBiomas) for four select municipalities along a gradient of human footprint show a range of land use/land cover types and configurations. Left panel illustrates the human footprint index (HFI) at the municipality level across Brazil, with focal municipalities outlined—Coromandel, HFI=12.5 (red), Barra do Garoas, HFI = 8 (orange), Uruara, HFI = 5.7 (green), Boca do Acre, HFI=2.5 (blue). Municipalities were chosen for illustration that fall below, at, and above key thresholds of HFI identified for different vector-borne diseases. The complexity and multidimensionality of land cover patterns is illustrated for each of these four municipalities along the HFI gradient in the maps on the right.

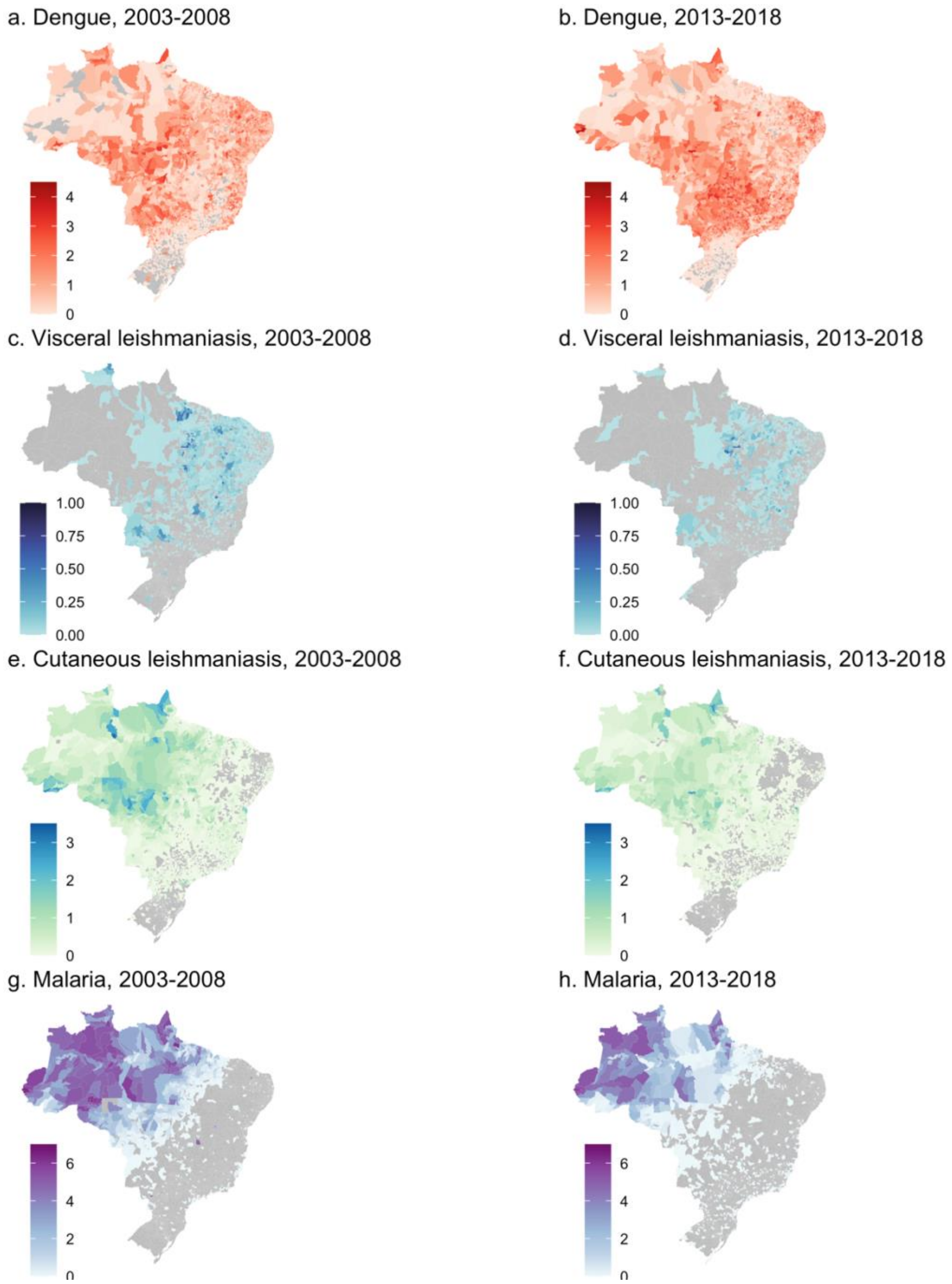

Supp. Fig. 2. Disease incidence (log-transformed average annual cases per 1000 per municipality) shifts in space between 2003-2008 (a,c,d,e) and 2013-2018 (b, d, f, h) for dengue (a,b), visceral leishmaniasis (c,d), cutaneous leishmaniasis (e, f), and malaria (g, h). Historical distributions of Zika and Chikungunya are not pictured as the first cases in Brazil were detected after 2013 and public reporting data starts in 2017 and 2016, respectively.

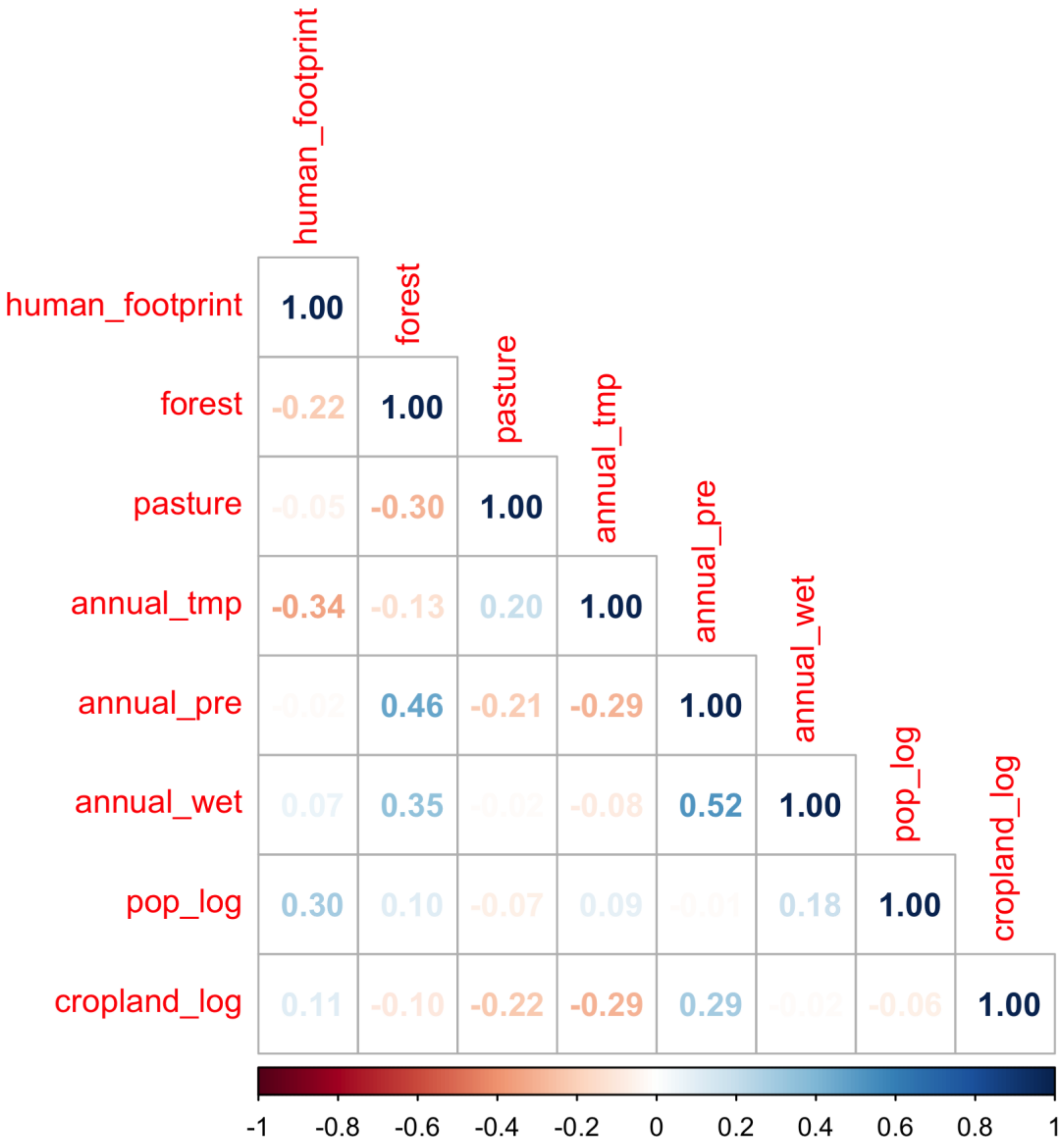

Supp. Fig. 3. Pearson correlation coefficients for covariates. Percentage pasture (pasture), annual mean temperature (annual_tmp), percentage forest cover (forest), annual mean precipitation (annual_pre), annual mean number of wet days (annual_wet), log percentage of cropland (cropland_log), log of population size (pop_log), human footprint index (human_footprint).

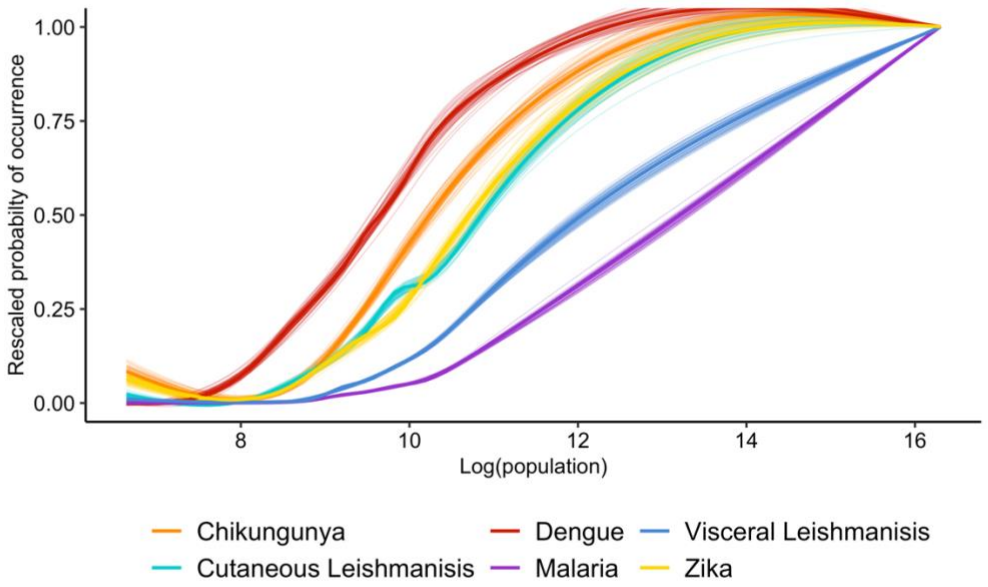

Supp. Fig. 4. Scaled partial dependence plots for log(human population size). Thin lines represent model output from each bootstrapped iteration, with thicker lines representing the mean value across the iterations.

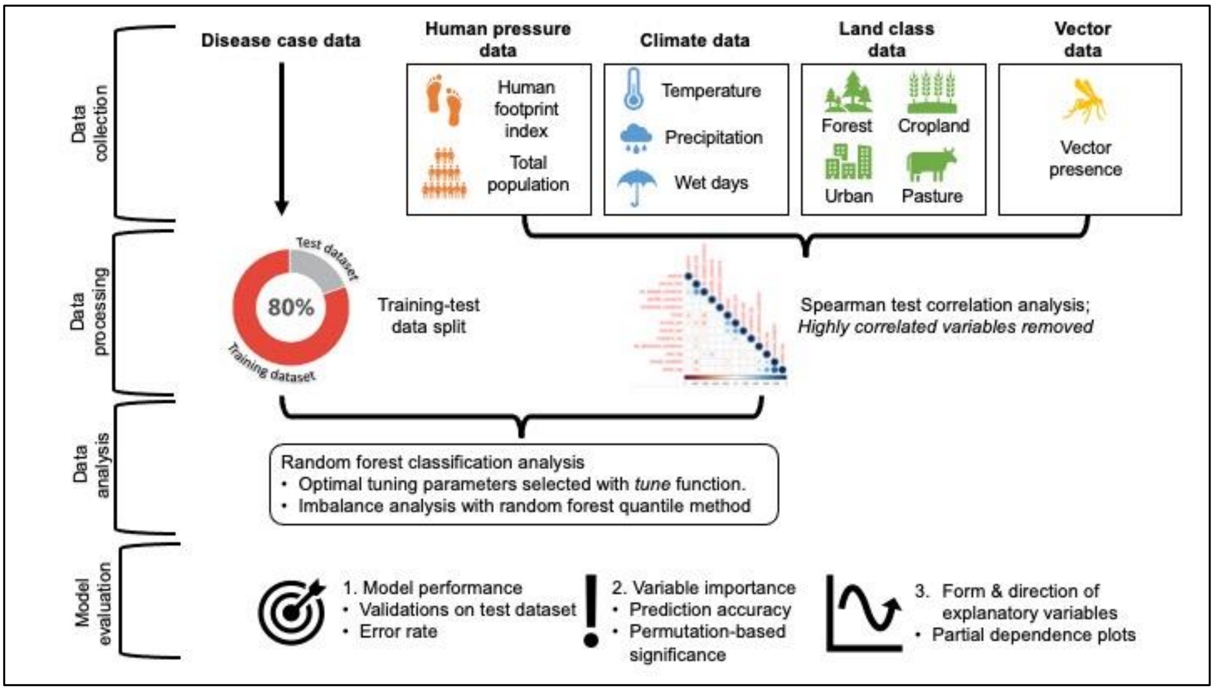

Supp. Fig. 5. Overview of analysis methods: following data collection, we split the response data into training and test subsets, removed highly correlated predictors, and used random forest classification analysis to identify predictors of occurrence and random forest regression analysis to identify predictors of incidence for each disease, then evaluated performance of all six models.

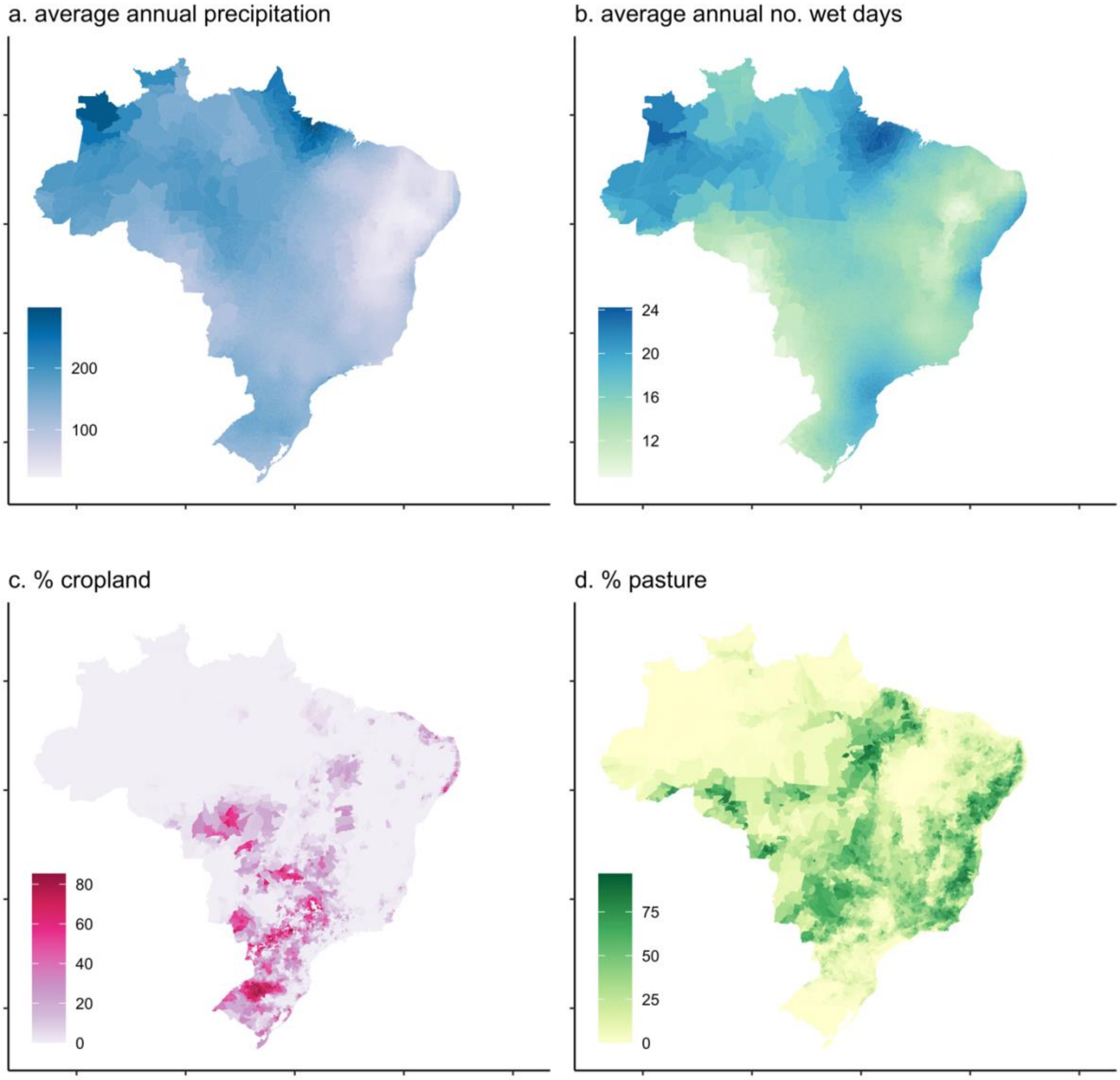

Supp. Fig. 6. Environmental covariates used to predict VBD occurrence in Brazil. a) Average annual precipitation (mm); b) average annual number of wet days; c) percentage of cropland; d) percentage of pasture.

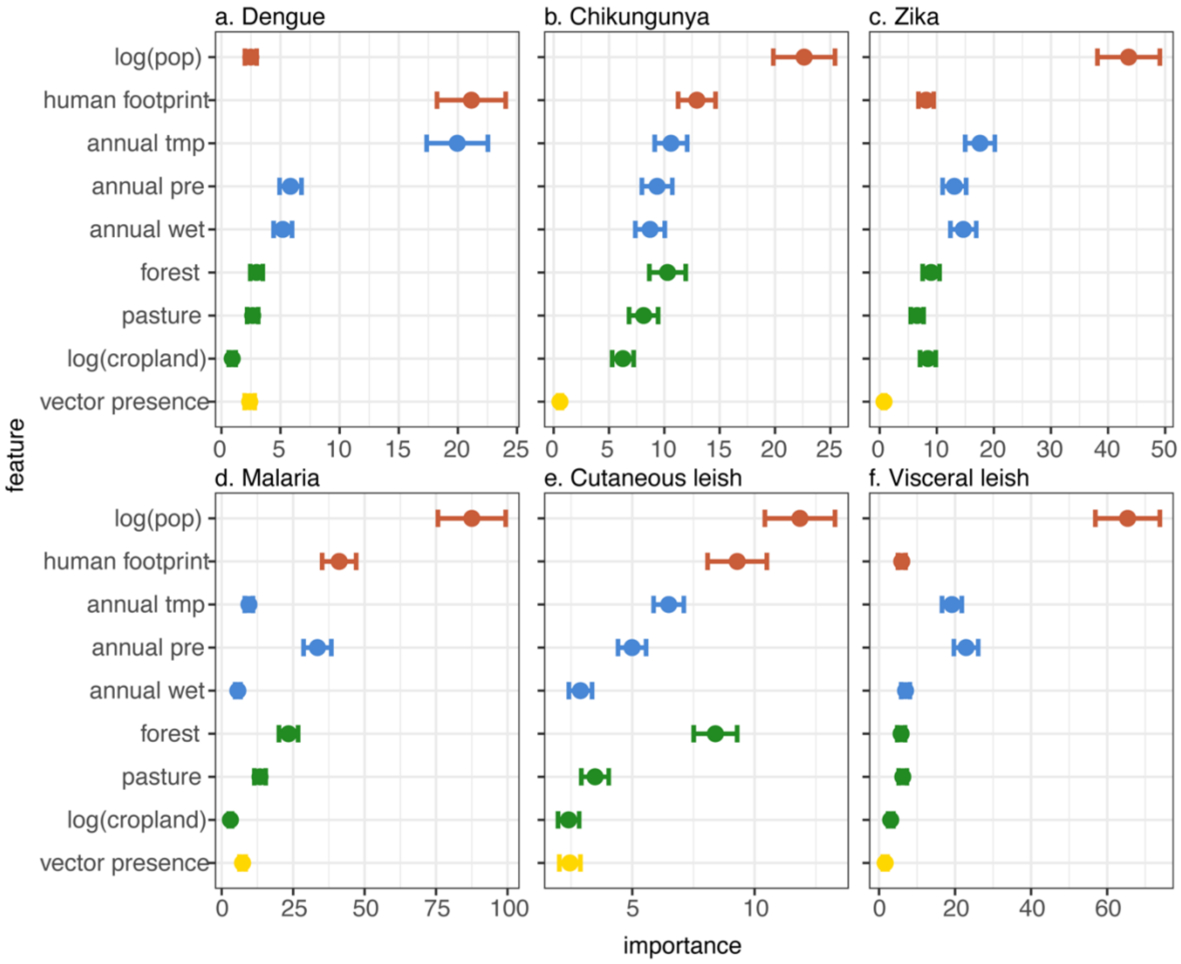

Supp. Fig. 7. Variable importance plots for models that included vector presence, in addition to covariates included in the primary models presented in the main text. Population, human footprint, climate, land cover, and vector presence variables were important for predicting the occurrence of a) dengue, b) chikungunya, c) Zika, d) malaria, e) cutaneous leishmaniasis, and f) visceral leishmaniasis. Importance values represent % increase in mean squared error when the focal variable is permuted. Log(population), human footprint index, annual temperature (ºC), annual precipitation (mm), % forest cover, % pasture cover, log(% cropland), vector presence. Variables are coloured by their category (red = anthropogenic, blue = climatic, green = land class).

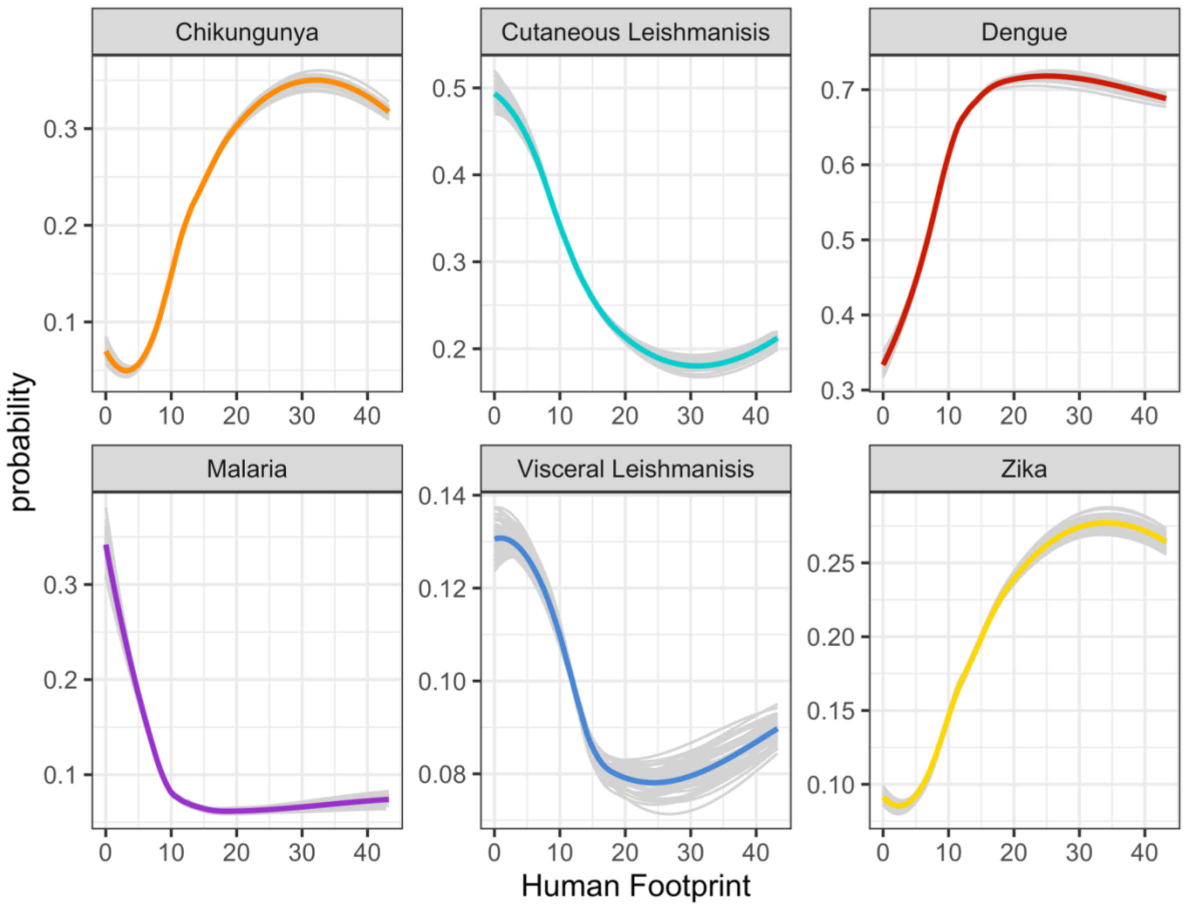

Supp. Fig. 8. Partial dependence plots for human footprint index (unscaled) showing probability of occurrence (y-axis) against human footprint index (x-axis) for all six focal diseases. Thin, grey lines represent model output from each bootstrapped iteration, while thicker coloured lines show the mean value across the iterations.

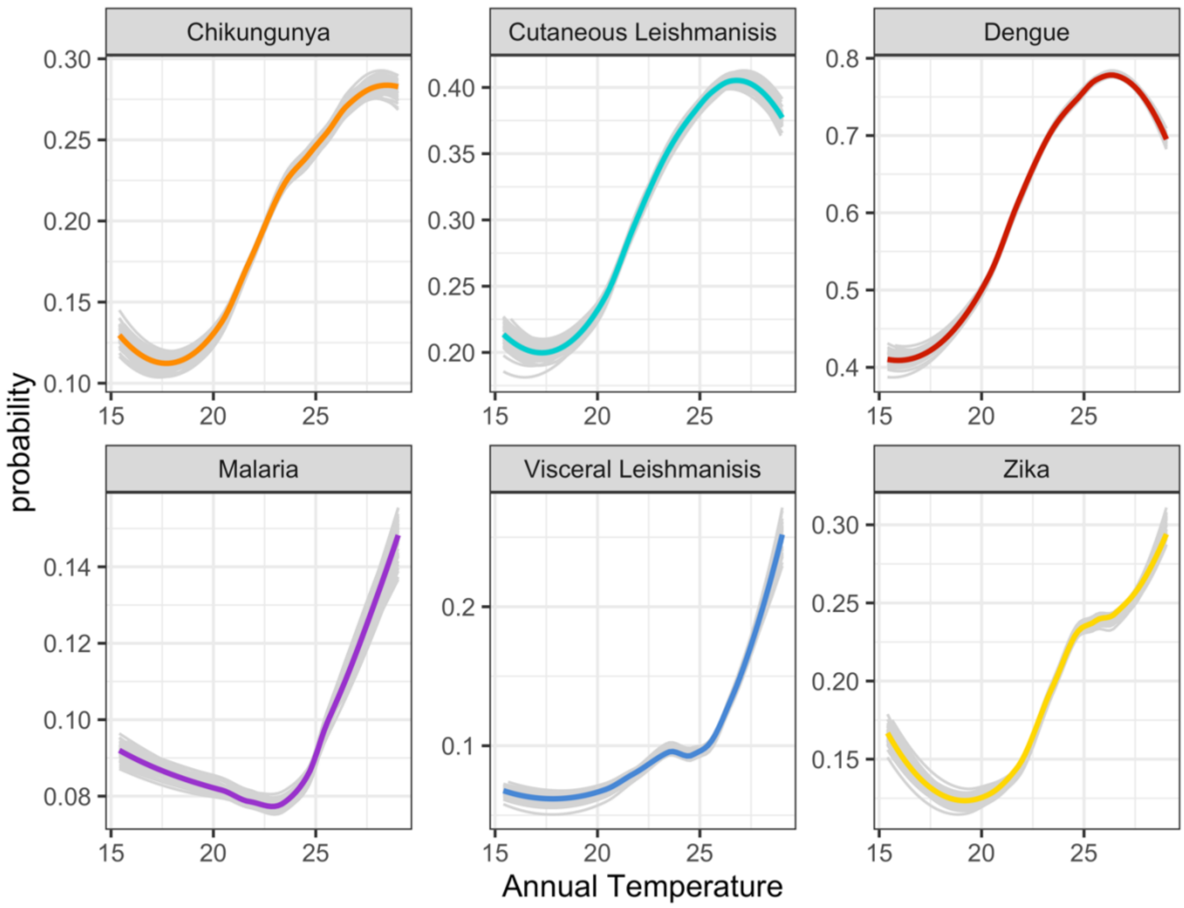

Supp. Fig. 9. Partial dependence plots for annual mean temperature (unscaled) showing probability of occurrence (y-axis) against annual mean temperature in ºC (x-axis) for all six focal diseases. Thin, grey lines represent model output from each bootstrapped iteration, while thicker coloured lines show the mean value across the iterations.

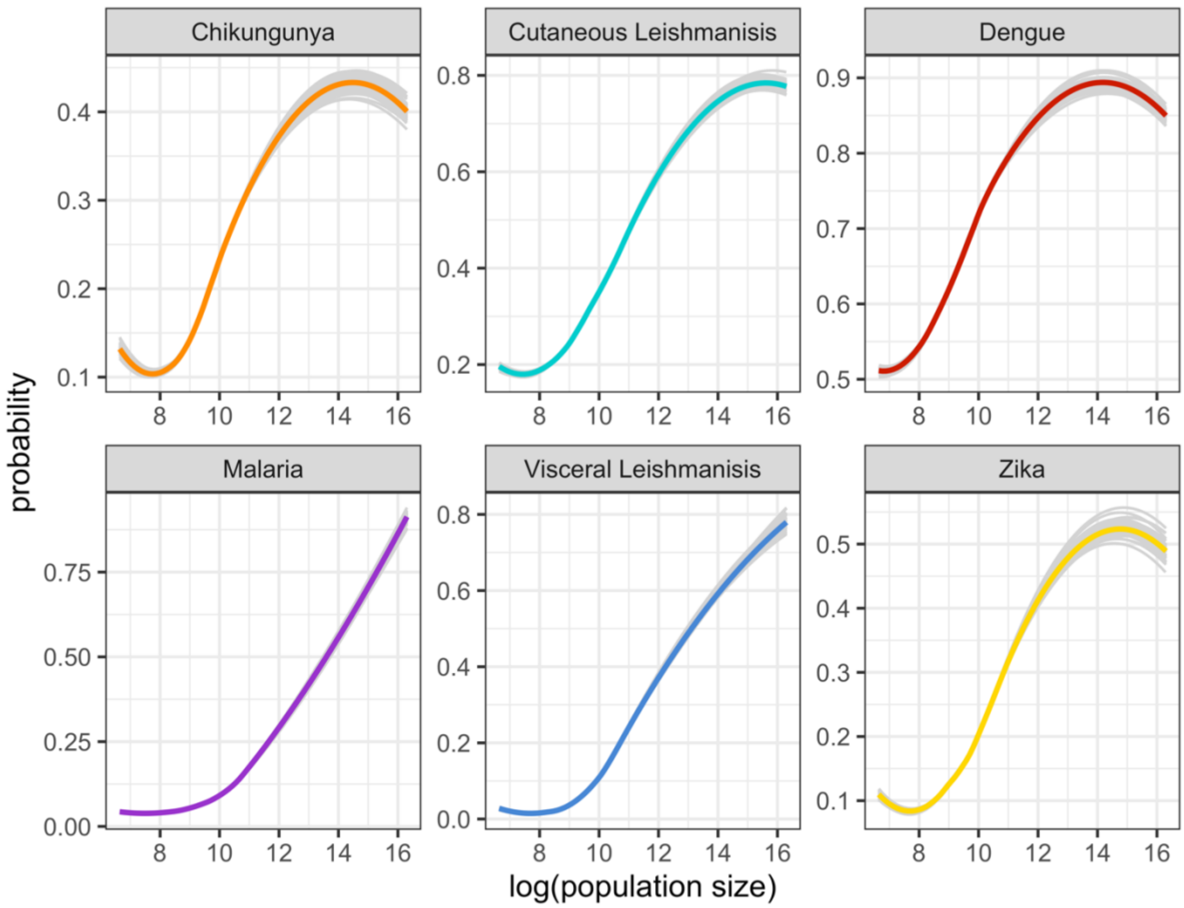

Supp. Fig. 10. Partial dependence plots for population (unscaled) showing probability of occurrence (y-axis) against log of human population size (x-axis) for all six focal diseases. Thin, grey lines represent model output from each bootstrapped iteration, while thicker coloured lines show the mean value across the iterations.

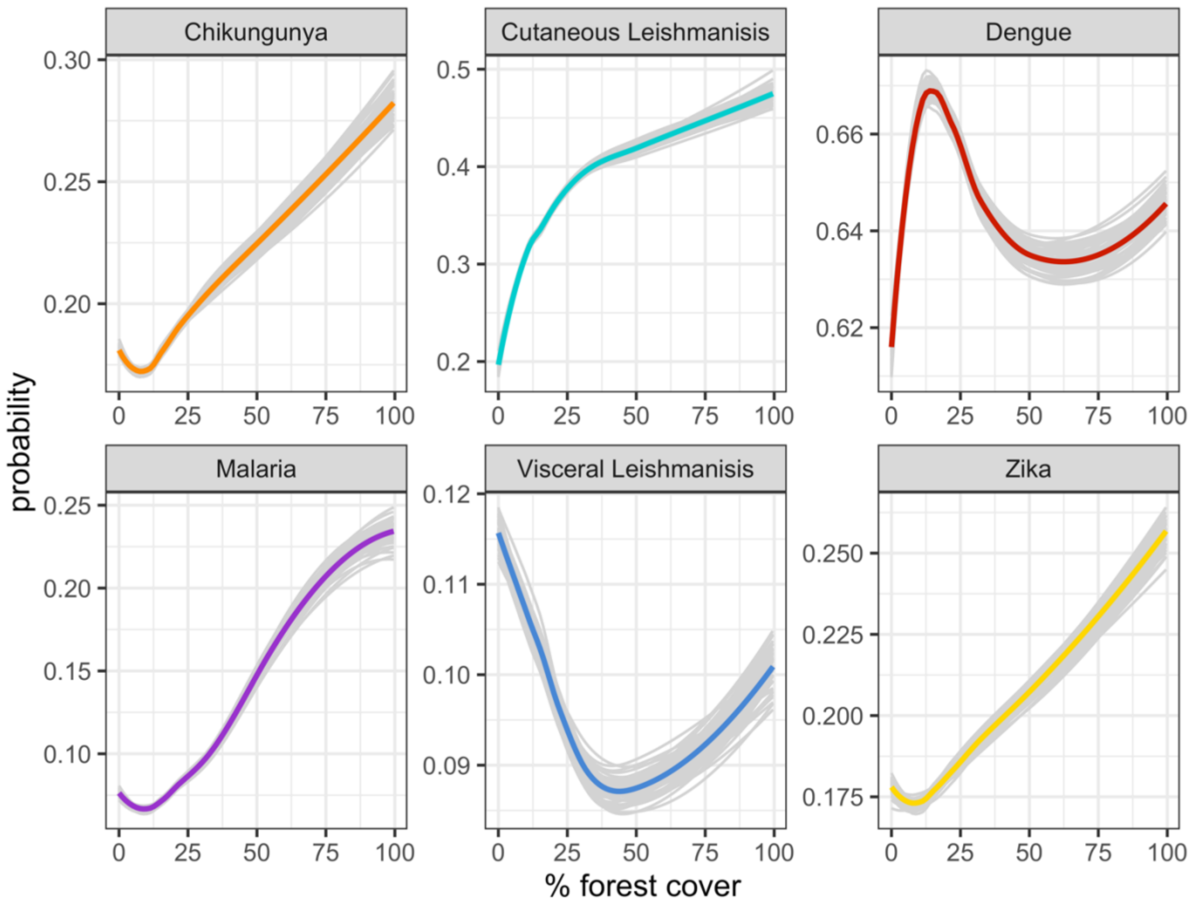

Supp. Fig. 11. Partial dependence plots for forest cover (unscaled) showing probability of occurrence (y-axis) against the percentage forest cover (x-axis) for all six focal diseases. Thin, grey lines represent model output from each bootstrapped iteration, while thicker coloured lines show the mean value across the iterations.
